# Green synthesis of silver nanoparticles from plant *Fagonia cretica* and evaluating its anti-diabetic activity through indepth *in-vitro* and *in-vivo* analysis

**DOI:** 10.3389/fphar.2023.1194809

**Published:** 2023-10-23

**Authors:** Haider Ali Khan, Mehreen Ghufran, Sulaiman Shams, Alam Jamal, Abbas Khan, Abdullah, Zuhier A. Awan, Mohammad Imran Khan

**Affiliations:** ^1^ Department of Biochemistry, Abdul Wali Khan University Mardan, Mardan, Khyber Pakhtunkhwa, Pakistan; ^2^ Department of Pathology, Medical Teaching Institution Bacha Khan Medical College (BKMC) Mardan, Mardan, Khyber Pakhtunkhwa, Pakistan; ^3^ Department of Biochemistry, Faculty of Science, King Abdulaziz University, Jeddah, Saudi Arabia; ^4^ Department of Chemistry, Abdul Wali Khan University Mardan, Mardan, Khyber Pakhtunkhwa, Pakistan; ^5^ Department of Environmental Science, Abdul Wali Khan University Mardan, Mardan, Khyber Pakhtunkhwa, Pakistan; ^6^ Department of Clinical Biochemistry, Faculty of Medicine, King Abdulaziz University, Jeddah, Saudi Arabia; ^7^ Centre of Artificial Intelligence for Precision Medicines, King Abdulaziz University, Jeddah, Saudi Arabia

**Keywords:** F. cretica, diabetes, oxidative stress, biogenic nanoparticles, AgNPs, Stz

## Abstract

One of the most widespread metabolic diseases, Type-2 Diabetes Mellitus (T2DM) is defined by high blood sugar levels brought on by decreased insulin secretion, reduced insulin action, or both. Due to its cost-effectiveness and eco-friendliness, plant-mediated green synthesis of nanomaterials has become more and more popular. The aim of the study is to synthesize AgNPs, their characterizations and further *in-vitro* and *in-vivo* studies. Several methods were used to morphologically characterise the AgNPs. The AgNPs were crystalline, spherical, and clustered, with sizes ranging from 20 to 50 nm. AgNPs were found to contain various functional groups using Fourier transform infrared spectroscopy. This study focuses on the green-synthesis of AgNPs from *Fagonia cretica* (*F. cretica*) leaves extract to evaluate their synthesized AgNPs for *in-vitro* and *in-vivo* anti-diabetic function. For the *in-vivo* tests, 20 male Balb/C albino-mice were split up into four different groups. Anti-diabetic *in-vivo* studies showed significant weight gain and a decrease in all biochemical markers (pancreas panel, liver function panel, renal function panel, and lipid profile) in Streptozotocin (STZ)-induced diabetic mice. *In vitro* anti-diabetic investigations were also conducted on AgNPs, comprising α-amylase, α-glucosidase inhibitions, and antioxidant assays. AgNPs showed antioxidant activity in both the DPPH and ABTS assays. The research showed that the isolated nanoparticles have powerful antioxidant and enzyme inhibitory properties, especially against the main enzymes involved in T2DM.

## 1 Introduction

Diabetes is associated with hyperglycaemia, insulin resistance, and excessive production of inflammatory markers. In turn, chronic hyperglycaemia produces metabolic impairment, oxidative stress, and kidney and liver damage (Souha et al., 2021). Worldwide, high co-morbidity rates can be attributed to the prevalence of type2 diabetes mellitus (T2DM), also known as Insulin Independent Diabetes Mellitus (IDDM). When pancreatic cells fail in T2DM, the liver, skeletal muscles, and adipose tissues become insulin resistant and insulinopenic, which causes metabolic dysfunction and organ failure in these organ systems. Pancreas secretes enzymes in response to signals, largely controlling blood glucose levels. The World Health Organization estimates that 422 million people worldwide currently suffer from diabetes, with 1.6 million fatalities attributable to the disease in 2016. This ranks diabetes as the seventh leading cause of morbidity and mortality worldwide. Worldwide, 640 million people will have diabetes by 2040 (Wahab et al., 2022). Reducing glucose absorption from the gastrointestinal system is one of the therapy methods for T2DM. In order to convert dietary carbohydrates into simple mono-saccharides, the enzyme α-glucosidase (also known as α-D-glucoside glucohydrolase) catalyzes the release of α-glucose from the non-reducing end of the substrate. Long-chain carbohydrates are also broken down metabolically by the α-amylase enzyme, which aids in their absorption in the gastrointestinal tract. The hydrolytic breakdown of oligosaccharides is reduced by inhibitors of these enzymes, delaying their absorption from the intestinal tract. One of the greatest ways to prevent the onset of late diabetes complications is to limit the postprandial rise in blood glucose, according to scientific research. In order to prevent T2DM and lower the after-meal glucose level in the body, these enzymes are crucial therapeutic targets (Ayaz et al., 2022). There are many types of drugs used to treat T2DM, such as sulfonylureas, α-glucosidase inhibitors, thiazolidine dione, repaglinide, and insulin therapies. However, the most generally used drug is “Metformin”, which has a lot of side effects and works slowly (Jackson et al., 1987).

The standard treatment for diabetes, metformin, indirectly stimulates adenosine monophosphate-activated protein kinase (AMPK) signalling pathway by inhibiting mitochondrial activity. Even though many people with T2DM prefer metformin, it may also reduce the intestinal absorption of vitamin B12 and induce flatulence, anorexia, diarrhoea, and stomach pain. In SH-SY5Y (cell-line) cells and in mice, AMPK is activated by being phosphorylated via the Calcium/calmodulin-dependent protein kinase kinase (CAMKK) pathway by AgNPs, which also raise the quantity of cytosolic calcium ions (Wahab et al., 2022). AMPK activation increases insulin sensitivity and could influence the effects of insulin by enhancing its activity (Li et al., 2019; Souha et al., 2021). Insulin attaches to its receptor, causing the phosphorylation cascade from insulin receptor substrate-1 (IRS1) to cause the transport of glucose into the cells. Increasing the protein levels of IRS1 will ultimately lessen the consequences associated with hyperglycaemia, as research has shown that animal models lacking IRS1 developed hyperglycaemia or T2DM. AgNPs cause an increase in IRS1 and glucose transporter 2 (GLUT2) expression, which lowers blood glucose levels. In addition, AgNPs increase insulin expression and secretion (Wahab et al., 2022).

Nanotechnology is currently a significant field since it involves the design, synthesis, and manipulation of particle structure, allowing it to be utilised in several fields, such as medicine/pharmacy and public health (Chandra et al., 2020; Mukhtar et al., 2020; Sabir et al., 2021; Sharma et al., 2021), cosmetics, food additives, agriculture (Behl et al., 2022), environmental protection (Nechifor et al., 2021) *etc.* Among numerous noble metal nanoparticles, AgNPs are of interest due to their electrical, unique optical and magnetic properties that allow them to be used in cosmetic products, composite fibres, electronic components, antimicrobial applications, biosensor materials and cryogenic superconducting materials, membranes (Nechifor et al., 2021; Dimulescu et al., 2021; Nechifor et al., 2022) *etc.*, The primary elements influencing the unique features of AgNPs are their size and form (15). In addition to this, they have been used in the diagnosis and management of a number of disorders (Shaik et al., 2018). AgNPs have been produced using a variety of physical, chemical, and biological processes. One of the widely used methods is chemical reduction, which employs a variety of inorganic and organic reducing chemicals as well as electrochemical processes and physicochemical reduction (Shukla and Makwana, 2014). Green synthesis, sometimes referred to as biosynthesis, is another method for producing AgNPs that uses algae, fungi, bacteria, or plant extract (Panigrahi, 2013; Ali et al., 2017).

Physical and chemical methods are less flexible, more expensive, and less environmentally beneficial than the green synthesis (Thomas et al., 2014). This method does not require a lot of solvent, high pressure, or high temperatures. Utilizing plant extracts has advantages over employing microorganisms, including easier access, fewer biohazards, and no special preservation of cell cultures (Pugazhendhi et al., 2016). AgNPs are largely utilized in electronic batteries, glass and ceramic pigments and medical devices to cure diabetes, malaria, cancer and tuberculosis. In addition to their catalytic characteristics, AgNPs are also employed in the detection of biological molecules (Asad et al., 2022). In recent years, antidiabetic, antibacterial, and anticancer properties of AgNPs have been demonstrated. It has been proposed that the antidiabetic activity of AgNPs is associated with the efficient inhibitory action of carbohydrate digesting enzymes such as α-amylase and α-glucosidase (Khodeer et al., 2023; Vesa and Bungau, 2023). Diabetes is now referred to be an oxidative stress-based condition since T2DM is also brought on by an imbalance between the cellular reactive oxygen species and the antioxidants produced by the body’s natural mechanisms. Apoptosis and maturation of β-cells rise as a result of high ROS generation, but insulin synthesis and secretion fall. Antioxidants are utilized to treat oxidative stress, and attention is shifting away from synthetic antioxidants toward natural antioxidants. Since they may easily penetrate deeply into the tissues, AgNPs provide a rich supply of antioxidants that are immediately available for action in the tissues. It has been demonstrated that AgNPs are an efficient scavenger of free radicals, particularly oxygen-based ones (Wahab et al., 2022). The antibiotic streptozotocin, which is generated by the bacterium *Streptomyces* achromogenes, is commonly used to cause diabetes in insulin-dependent as well as non-insulin-dependent animals. The reason STZ causes diabetes is that it is employed preferentially for the degeneration of insulin generating β-cells, which ultimately leads to the development of necrosis. Its activity on the β-cells of the pancreas is related with characteristic alterations in the blood glucose concentration and insulin level. These changes reflect defects in the beta cell’s ability to operate normally. The glucose oxidation process is altered by STZ, and insulin production and secretion are both reduced. Because it causes DNA damage and death in beta and alpha cells of the pancreas, it is highly damaging to the pancreatic beta and alpha cell populations. The kidney and liver are also sensitive to the toxicity of STZ, just like the beta- and alpha-cells in the pancreas. (Khan et al., 2022). STZ induces diabetic mice better than Alloxan due to its higher inductive price and lower toxicity. STZ induces DM2 better than nicotinamide. Alloxan kills quickly and reduces bodyweight. This article shows that STZ is safer and easier to use than Alloxan for T2DM (Islam and Code, 2017).

Preliminary pharmacological tests have shown that the plant *F. cretica* has significant therapeutic potential, making it one of the plants with a strong therapeutic value that is highly pursued by scientists. The green, upright, tiny, prickly bush known as *F. cretica* L. is typically found in the arid calcareous rocks of Pakistan and western India, in addition to Morocco, Tunisia, Algeria, Egypt, Cyprus and Saudi Arabia. Both modern science as well as traditional medicine tout the healing powers of Fagonia species, noting their profound therapeutic effects against a wide range of illnesses. It has a sour and bitter flavour and has been shown to be effective in treating liver, blood, brain, and inflammation-related disorders.

Extracts from the Fagonia genus are used for their wide range of medicinal properties, including those of diuretic, stomachic, antipyretic, anti-dysenteric, antiasthmatic, antitumor, tonic, analgesic, bitter, analgesic and stimulant (Kiani et al., 2022). Considering the benefits of green synthesis over other methods, a new plant extract was found out called *F. cretica*, as a reducing also stabilising agent for the synthesis of AgNPs. This is because *F. cretica* is abundant and cheap, and it does not involve any fungal or bacterial species like other biosynthesis methods do. This eliminates all clinical concerns that come with other biological synthesis methods, making *F. cretica* extract a good choice (Zulfiqar et al., 2019).

The current research focuses on the biosynthesis of AgNPs from *F. cretica* and characterizations using ultraviolet-visible (UV–Vis) spectroscopy, scanning electron microscopy, transmission electron microscopy and Fourier transform infrared (FT-IR) spectroscopy. Furthermore, AgNPs of *F. cretica* will be tested for its anti-diabetic effects by *in-vitro* assays (α-amylase, α-glucosidase and anti-oxidant assays) as well as *in-vivo* studies on mice that have been made diabetic by injection of STZ.

## 2 Materials and methods

### 2.1 Plant collection

The leaves of *F. cretica* were collected from the fields around the Mardan region (Pakistan). The leaves of *F. cretica* were identified and authenticated by Dr. Mohib Shah in the Department of Botany, Faculty of Chemical and Life Science, Abdul Wali Khan University Mardan, Khyber Pakhtunkhwa, Pakistan. The leaves of *F. cretica* specimen were deposited in the institutional herbarium with voucher number AWKUM. BOT.32.2.18. The *F. cretica* leaves were washed thoroughly with water and then with deionized water. The leaves were thinly cut into small pieces and shade-dried at room temperature.

### 2.2 Preparation of *F. cretica* leaves extract

After being washed with water to remove dust and other chemicals, the fresh *F. cretica* leaves were air dried to remove any remaining moisture or wetness before being milled into a powder. For the plant extract, 160 mg of *F. cretica* was combined with 10 mL of distilled water in a 250 mL conical flask and boiled for around 10–15 min. After that, Whatman filter paper was used to filter the resulting solution. The solution was then stored at 4°C for subsequent use in the process of transforming silver ions (Ag+) into AgNPs (Ali et al., 2016).

### 2.3 Silver nitrate (AgNO_3_) salt synthesis

In order to make 5 mM of AgNO_3_, 85 mg of AgNO_3_ (Sigma-Aldrich, St. Louis, MO, United States of America) was dissolved in 100 mL of distilled water with vigorous stirring for about 30 min at room temperature (Kwon et al., 2005).

### 2.4 The synthesis of AgNPs

To adjust the pH of silver nitrate solution to 11, 1 millimole of NaOH was added very slowly. The silver nitrate solution was then added to the extract while it was still being agitated. A change in the solution’s colour indicates the formation of AgNPs, which may be observed under a microscope. Once the nanoparticles were made, they were centrifuged at 20,000 RPM to separate them from the aqueous phase and then rinsed again. A 7-pH was achieved by centrifuging and washing the nanoparticles. Finally, the synthesised AgNPs were dried in a vacuum oven at 80°C (Jacob et al., 2012; Nikbakht and Pourali, 2015).

### 2.5 Characterization of AgNPs

#### 2.5.1 UV-visible spectroscopy analysis of AgNPs

AgNPs were produced utilising *F. cretica* leaves extract as reducing agents, and the AgNO_3_ solution was validated using UV-vis spectroscopy. The absorbency of the synthesized AgNPs were evaluated using distilled water as a control (blank). To investigate the synthesis of AgNPs, UV-vis spectroscopy was done using a UV-1602 double beam UV-vis spectrometer (Lambda 25, PerkinElmer, Waltham, MA, United States) with a resolution of 1 nm. AgNPs produced from the reaction mixture were tested for their spectrum absorbency at wavelengths ranging from 200 nm to 800 nm.

#### 2.5.2 Scanning electron microscopy

To describe the shape of AgNPs, scanning electron microscopy (SEM) was employed. The leaves extract of *F. cretica* were used to prepare AgNPs, which were properly air-dried at 35°C with the use of a vacuum dryer. A small droplet of the AgNPs was then dropped on a carbon-coated SEM grid and left to dry. The sample was subsequently silver coated using a spi-module sputter coater (Auto fine coater) at the University of Peshawer, and morphological features were examined using Field-Emission scanning electron microscopy (FE-SEM) (JSM-5910-JEOL) (Lab tech., Yokosuka, Japan).

#### 2.5.3 Transmission electron microscopy

Transmission electron microscopy (TEM) was used to examine the morphology and size of the AgNPs. The ultra-high-resolution transmission electron microscope has a voltage of 200 kV. (JEOL, Model No. JEM 2100 HR with EELS). The 5 μL of the AgNP solutions were applied on copper grids that had carbon coatings to create the TEM grids, which were then dried under a light.

#### 2.5.4 Fourier-transform infrared (FT-IR) spectroscopy

To make the sample pellet, the AgNPs were dried, mixed with KBr, and pressed using a hydraulic pellet press. FT-IR spectroscopy was performed on the sample at the “Department of Chemistry”, “Bacha Khan University, Charsadda, Pakistan”, using the “PerkinElmer spectrometer FT-IR SPECTRUM ONE” (Spectra Lab Scientific Inc., Markham, ON, Canada) with a resolution of 4 cm^*−*1^ in the range of 4,000 cm^*−*1^ *–*0 cm^*−*1^

### 2.6 *In vitro* studies

#### 2.6.1 Assay for α-glucosidase inhibition

The samples were tested for α-glucosidase inhibition using (Mccue et al., 2005). In short, 0.5 units/mL of the α-glucosidase enzyme was dissolved in 0.1 M phosphate buffer to make a solution (pH 6.9). The final enzyme mixture contains 120 uL of 0.1 M phosphate buffer and 20 uL of α-glucosidase (0.5 units/mL). In the same buffer, a solution of 5 mM p-Nitrophenyl—D-glucopyranoside, which is a substrate, was made (pH 6.9). Test samples with concentrations between 31.25 μg/mL and 1,000 μg/mL were prepared and combined with enzyme solution. The samples were then incubated for 15 min at 37°C. Finally, 20 μL of substrate solution was added to the enzyme mixture, which was then put back in the 37°C incubator for 15 min. When 80 μL of 0.2 M sodium carbonate solution was added, the reaction was done. UV visible spectrophotometer was used to measure absorbance at 405 nm (Thermo electron corporation United States). The part of the system that did not have α-glucosidase was used as a blank, and acarbose was used as a positive control. Each experiment was done three times, and the percent inhibition was figured out with a formula.

$$\%\text{ Inhibition}=\left[\frac{Absorbance\;of\;Control-Absorbance\;of\;Sample}{Absorbance\;of\;Control}\right]\times 100$$

#### 2.6.2 Assay for α-amylase inhibition

The α-amylase inhibitory experiments followed the same protocol (Nair et al., 2013). To summarize, plant extracts (test substances) in concentrations ranging from 31.25 μg/mL to 1,000 μg/mL were combined with 20 μL of enzyme in 200 μL of 0.02 M sodium phosphate buffer. After 10 minutes at 25°C ± 3°C, 200 μL of starch was added to the assay mixes. The 400 μL of DNS reagent (dinitro salicylic acid) was added to the mixture to stop the process. After 5 minutes in a boiling water bath, the resulting solution was chilled. Once the liquid had cooled, further to dilute it, 15 mL of distilled water was added, and the absorbance at 540 nm was recorded. Acarbose was the standard drug, and enzyme inhibition was calculated with the help of a formula.

$$\%\text{inhibition}=\left[1-\left(A/B\right)\right]\times 100$$

Where A = absorbance of test and B = absorbance of enzyme control.

#### 2.6.3 DPPH antioxidant assay

The DPPH (α, α-diphenyl-β-picrylhydrazyl) anti-radicals study was carried out in accordance with the protocol we previously published (Boly et al., 2016; Ovais et al., 2018). Initially, a 0.1% methanolic-DPPH solution was prepared and 100 μL of it was combined with the same volume (100 μL) of test samples in 96-well plates, followed by 30 minutes incubation in a dark place 25°C ± 3°C. The offered samples’ solutions were produced in concentrations ranging from 1,000 to 62.5 μg/mL. Similarly, Trolox methanolic solution (100 μM) was made, followed by its five concentrations/dilutions of 1,000, 500, 250, 125, and 62.5 μM. Following incubation, a decrease in DPPH colour was noticed, and absorbance at 540 nm was measured using a microplate reader (Chen et al., 2013). The percentage of scavenging was calculated as follows:

$$\%\text{ Scavenging}=\left[\left(A0\;–A1\right)/\;A0\right]\times 100$$

#### 2.6.4 ABTS antioxidant assay

The study of ABTS (2,2′-azino-bis(3-ethylbenz-thiazoline-6-sulfonic acid) antiradicals was conducted according to the protocol we had previously reported (Ayaz et al., 2017; Mir et al., 2019). 192 mg of ABTS salt were dissolved in distilled water to create the ABTS solution, which was then transferred to a 50-milliliter flask and the volume was adjusted. Following the addition of 1 mL of the preceding solution to 17 μL of 140 mM potassium persulphate, the mixture was left in the dark for 24 h.

Methanol was used to dilute 1 mL of the reaction mixture to 50 mL to generate the final ABTS dilution for the test. Consequently, 190 μL of ABTS solution was added to 10 μL of sample solutions and 96-well plates, which were then incubated for 2 hours at room temperature in a dark place. After incubation, the decrease in ABTS colour intensity was measured using a microplate reader at 734 nm. At concentrations between 1,000 and 62.5 μM, Positive control solutions were created (Ayaz et al., 2022). The percentage of ABTS scavenging was calculated using the following equation:

$$\%\text{ Inhibition}=\left[\frac{\mathrm{Absorbance}\;\mathrm{of}\;\mathrm{Control}-\mathrm{Absorbance}\;\mathrm{of}\;\mathrm{Sample}}{\mathrm{Absorbance}\;\mathrm{of}\;\mathrm{Control}}\right]\times 100$$

### 2.7 *In vivo* anti-diabetic activity

#### 2.7.1 Experimental animals

Male Balb/C albino mice that were 6 weeks old and weighed between 25 and 35 g were kept in separate cages at 22°C–26°C with a 12-h light/dark cycle and free access to standard lab food. This study was approved by the Institutional Review Board of the Department of Biochemistry, Abdul Wali Khan University Mardan. The project identification code of the present study is Awkum/Biochem/2021/14, dated 10^th^ February, 2021.

#### 2.7.2 Acute toxicity study

According to the recommendations made by the Organization for Economic Cooperation and Development (OECD), an acute toxicity test was carried out (Leist et al., 2012)**.** Twenty Balb/C albino mice were used in the experiment, and they were split into four groups of five animals each. A single dosage of silver nanoparticles (AgNPs) was administered to the mice in each group. The concentrations were 5, 10, 15, and 20 mg/kg body weight for groups 1–4, respectively. The mice had been observed for 30 min after the AgNPs were given in order to identify any immediate effects, such as mortality or abnormalities in behavior. The mice were then routinely observed at 4 and 24 h to look for any additional behavioral or symptom changes.

#### 2.7.3 Induction of T2DM

The mice were fasted overnight before receiving a single intraperitoneal (i.p.) dose of freshly prepared STZ (45 mg/kg body weight) in citrate buffer (0.05 M) to cause diabetes in the test animals. Then, 5% glucose solution was administered via gavage. While other groups received injections of STZ, the control group received only injections of citrate buffer. By calculating the blood glucose level 72 h after receiving a STZ injection, diabetes was artificially induced and confirmed. Further diabetic studies used mice whose blood glucose levels were higher than 180 mg/dL. As a standard medication, metformin (200 mg/kg) was used. Body weight, random-fed blood glucose levels, and fasting blood glucose levels were used to evaluate the anti-diabetic activity. Mice were divided into the four groups listed below (*n* = 5).

#### 2.7.4 Experimental groups

Male Balb/C albino mice were randomised into four groups. The control group, i.e., first group received water daily for 21 days 15 mice in the second group were injected with 45 mg/kg STZ i. p. to induce diabetes for 5 days. They were then divided into three more groups: diabetics who received no treatment, diabetics who received AgNPs from the *F. cretica* plant, and diabetics who received metformin. All groups received 21-day treatment. Five mice per group were evenly distributed.

• Group I: (Normal control group) Water was injected i. p. into the control group’s (n = 5) mice.
• Group II: (STZ-treated Control group): For 21 days, distilled water was given to diabetic mice.
• Group III: AgNPs treated group: Diabetic mice were administered an i. p. injection of AgNPs.
• Group IV: Mice with diabetes were given daily doses of metformin (200 mg/kg body weight) for 21 days.

A weighing balance was used to determine the body weight of all of the experimental mice. From the beginning to the end of the study, the weight of the animals was regularly monitored (1st and 21st day). Blood glucose levels were measured using the “Rupturing tail vein technique” using the “EASYGLUCO” metre on the first, seventh, 14th, and twenty-first days of treatment.

### 2.8 Biochemical evaluation

The blood samples were sent to a medical diagnostics laboratory so that blood biochemical parameters could be measured. Mice were given anesthesia before they were euthanized for the experiment’s completion, and blood was taken through cardiac puncture. Blood samples were kept in centrifuge tubes chilled to −20°C, while tissue samples were kept at −70°C on ice. Total cholesterol (TC), triglyceride (TG), high density lipoprotein (HDL), low density lipoprotein (LDL), liver functional markers such as serum alanine transaminase (ALT), alkaline phosphatase (ALP), serum bilirubin, serum albumin, and renal functional markers such as serum urea, uric acid, creatinine, and amylase were measured in the separated serum after centrifugation at 6,000 rpm for 10 min using spectrophotometer (MINADRY BA88A, Shenzhen Mindray Bio-Medical Electronics Co., Ltd. Mindray Building, Keji 12th Road South, Hi-tech Industrial Park, Address: Nanshan, ShenZhen518057, P.R. China).

### 2.9 Histological examination of pancreas

To determined the effect of biosynthesized AgNPs, we have performed the histological analysis of normal, STZ induced diabetic, AgNPs and Metformin tissue. The pancreas of all groups were preserved in 10% formalin followed by dehydration in 70–100% ethanol. The tissue were cleared in xylene and embedded in paraffin. Sample were cut with microtome followed by staining as per protocol (Bancroft and Gamble, 2008). The tissue were observed under Nikon microscope (Nikon Y-THM Japan-0122,604; Nikon ECLIPSE 50i Nikon Corporation, Japan).

### 2.10 Statistical analysis

All the *in-vitro* tests were performed three times, and the collected data were presented as Mean ± Standard Error of the Mean (SEM). One-way ANOVA accompanied by multiple comparison, for the statistical comparison of test samples to control groups, Dunnett’s test was utilized. The data of *in-vivo* were analysed with the Origin v19 software, and a one-way analysis of variance (ANOVA) was used, followed by the Tukey’s HSD *post hoc* test for multiple comparisons. Post hoc analysis using the least significant difference test was performed for intergroup comparisons. *p*-values less than 0.05 were considered statistically significant.

## 3 Results

### 3.1 Plant extract preparation

Following the manufacture of the *F. cretica* extract solution, the silver nitrate salt solution was created by dissolving 5 mM silver nitrate salt in distilled water (100 mL). This plant is renowned for its therapeutic properties. The subject plant was found to be useful for treating urinary discharges, tumours, stomach problems, scabies, fever, and toothaches (Saleh et al., 2011). It was also noted to have antibacterial, anti-inflammatory, anti-hemorrhagic, thrombolytic, and antioxidant characteristics (Sajid et al., 2011) and anti-diabetic actions (Kamran et al., 2017).

### 3.2 Green synthesis of AgNPs

After the preparation of solutions containing silver nitrate salt and plant extract from *F. cretica*, both solutions were combined in the correct proportions. The 5 mL aqueous *F. cretica* extract was correctly combined with 45 mL of 5 mM AgNO_3_, and the mixture was allowed to stand until the solution’s colour changed from yellow to blackish brown. For an aqueous solution mixture containing 5 mM silver nitrate, gradual colour shifts from yellow to reddish yellow and from reddish yellow to dark brown were seen. The entire production of AgNPs is indicated by the complete transformation of the aqueous solution from yellowish brown to blackish brown (AgNPs). Metal nanoparticles are responsible for these unique colour shifts (Johnson et al., 2014). A centrifugation process was used to separate the AgNPs from the prepared solution after their production. For approximately 40 min, the AgNPs solution was centrifuged at a maximum speed of 6,000 rpm. After being formed into a pellet, the AgNPs were rinsed with distilled water three times. AgNPs powder was produced by drying the extracted pellet in an oven at a temperature at 60°C. AgNPs powder was obtained after 24 h of drying time. Reducing Ag^1+^ to Ag^0^ causes the colour change (Yasmin et al., 2020). Free silver atoms (Ag^0^) formed AgNPs by nucleation and growth (Raza et al., 2016). SPR (surface plasmon resonance) is caused by electron oscillations in the conduction band. UV-visible absorption monitored the colour shift. Chemical stability and medicinal uses make AgNPs clinically relevant. AgNPs are made using chemical or physical processes. Biosynthesis approaches, which use plant extracts to reduce NP metal salts, are popular due to their simplicity, low cost, and safety (Souha et al., 2021).

### 3.3 Characterization of AgNPs

**FIGURE 1:**
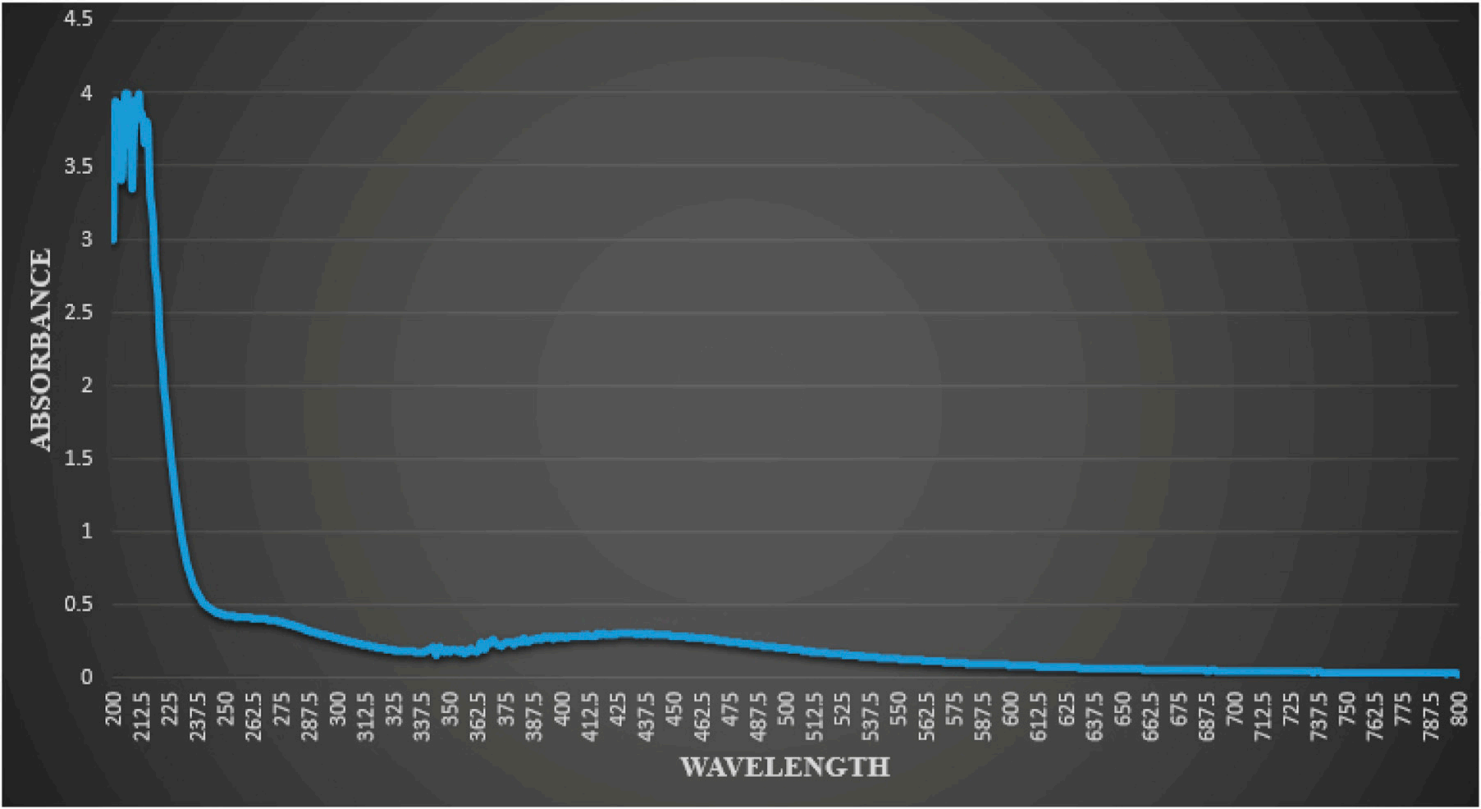
Result of UV-vis spectrophotometric analysis of reaction mixture indicating typical absorbance peak of AgNPs, showing peaks at (205.5 nm, 206.5 nm and 211.5 nm).

**FIGURE 2:**
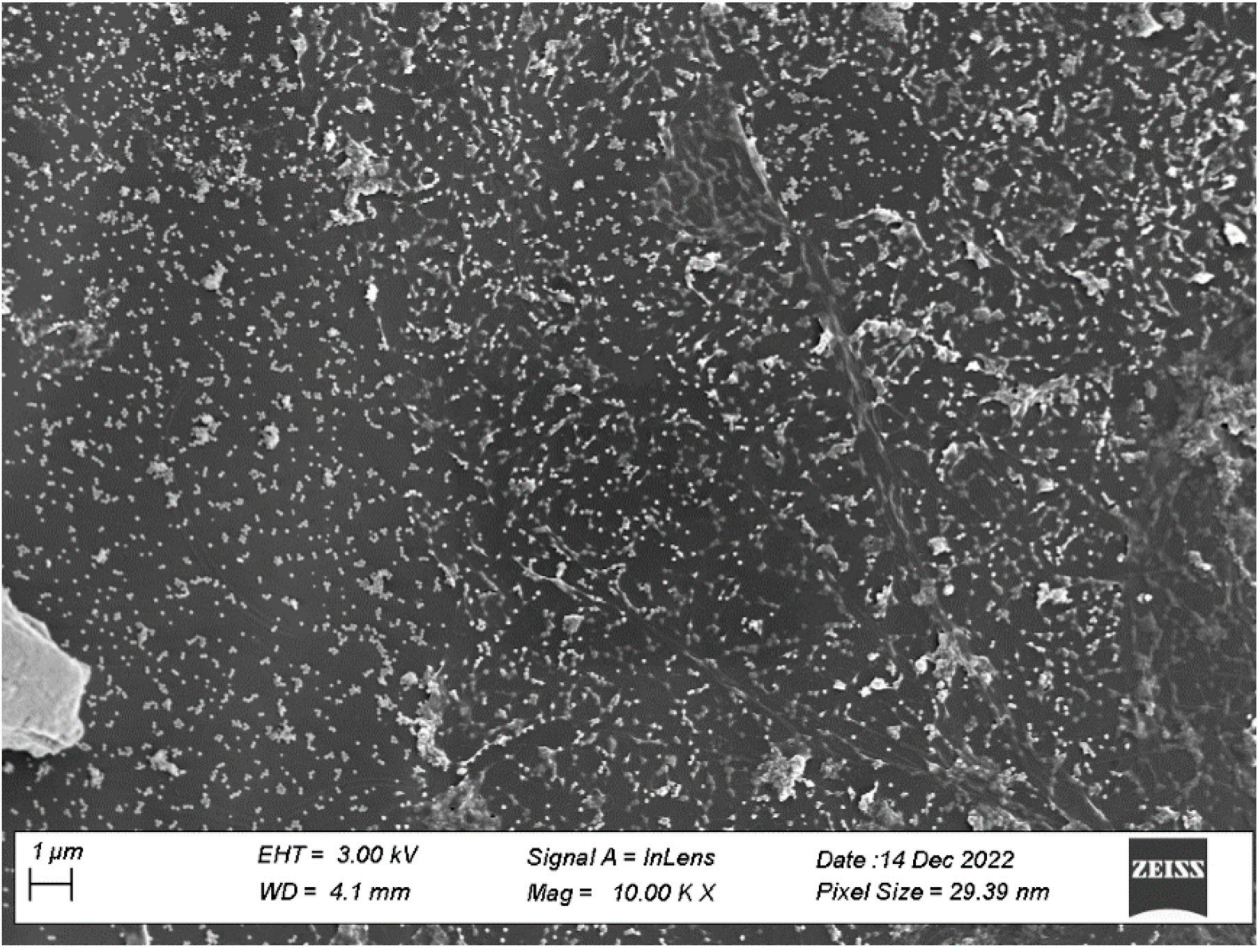
SEM image of AgNPs of *F. cretica*. The shape and size of the AgNPs were measuredusing SEM.

**FIGURE 3:**
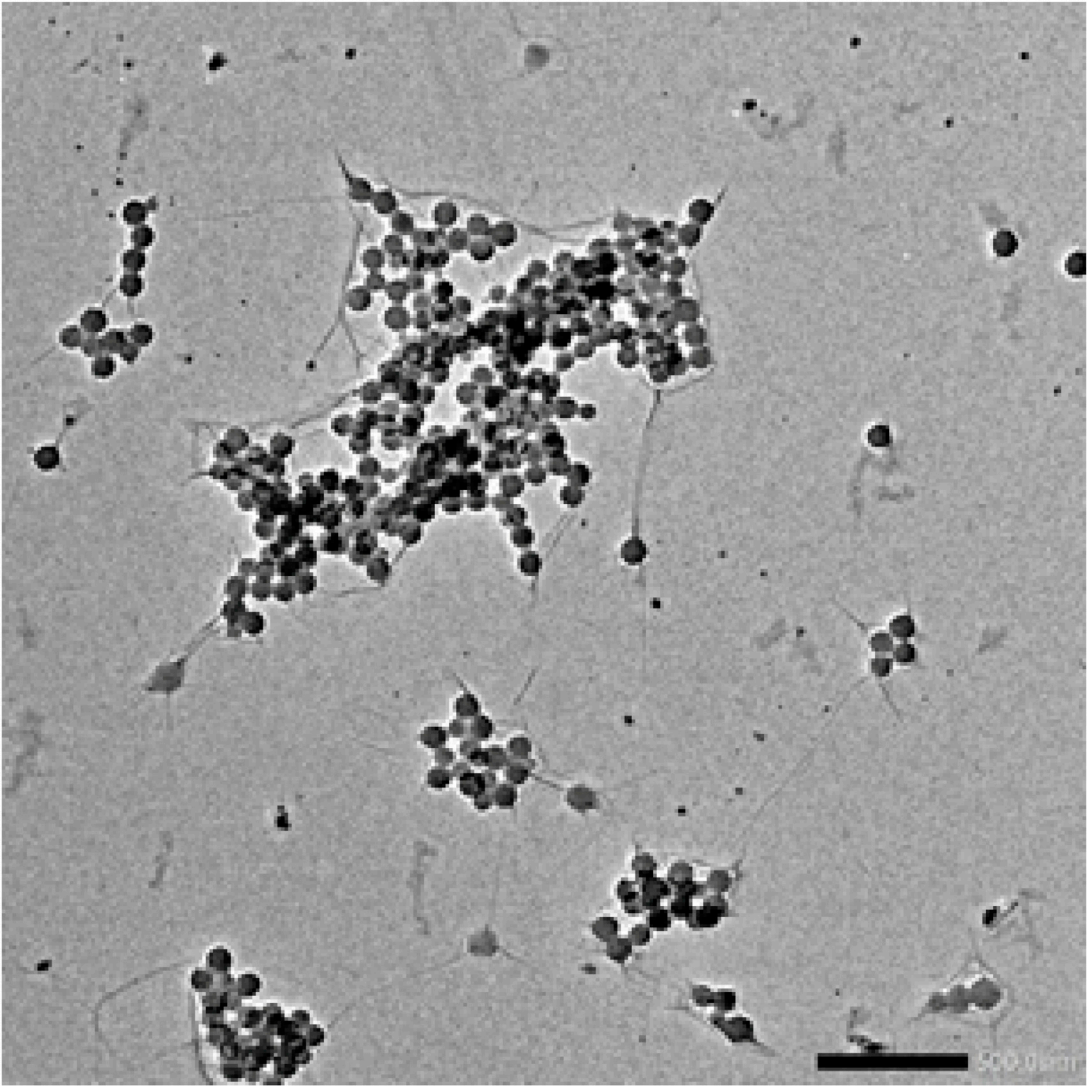
TEM image showed AgNPs of *F. cretica* werespherical in shape.

Characterization of synthetic AgNPs was done before the anti-diabetic properties of AgNPs were examined. To put it briefly, this study involved the room-temperature green synthesis of AgNPs using *F. cretica* leaf extract. Upon the reaction of the plant extract with the silver nitrate solution, the mixture’s visual appearance changed to brown, indicating the creation of AgNPs. UV-Vis spectroscopy, SEM, TEM, and FT-IR were used to characterise biosynthesized AgNPs. A high broad absorbance peak was seen in the UV-Visible spectra at 205.5, 206.5, and 211.5 nm, confirming the production of AgNPs (Figure 1). FESEM was used to examine the morphology of AgNPs, and it was discovered that the produced nanoparticles were spherical in shape and had a size of 29.39 nm (Figure 2). Previous research used FESEM to examine the morphology of AgNPs, and it was discovered that the produced nanoparticles were spherical in form and ranged in size from 20 to 50 nm (Bibi et al., 2019). The TEM picture displayed in Figure 3 clearly shows that the AgNPs were spherical in shape. The majority of the biosynthesized AgNPs’ TEM and HRTEM images revealed that the particles were almost spherical in shape and crystalline in form. In particular, the HRTEM images revealed the presence of lattice fringe that correlates to Ag plane (Soman and Ray, 2016).

**FIGURE 4:**
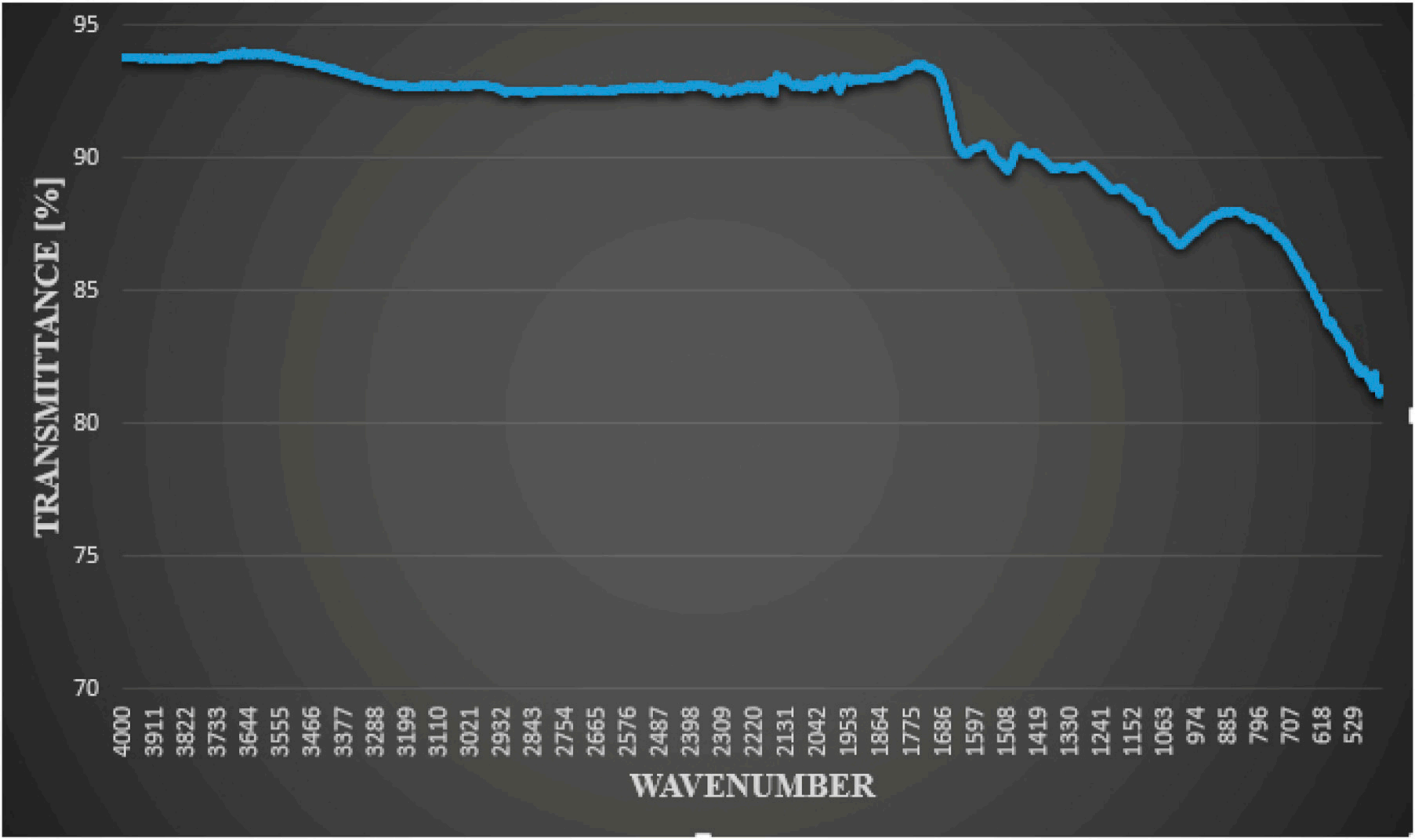
FT-IR analysis of green-synthesized AgNPs by reaction of silver nitrate with leaves extract of *F. cretica* showed functional groups.

To examine the function of the functional groups in the plant extract as a capping agent and bio-reduction agent, FT-IR analysis was done (Kumar et al., 2018). Figure 4 shows the FT-IR absorption spectra of green AgNPs. The FT-IR analysis’s measured spectra were compared to Coates’s previously reported reference value (Coates, 2000). The leaf extract of *F. cretica* showed C-H bending at the peak values of 611.01 and 623.73, indicating the presence of carbohydrates. The aromatic C-H bending caused by glycosides was revealed by the absorption peak at 721.87. Peak at 978.33 suggested a C=C stretch, which may have been caused by the plant’s coumarin, tennins, and terpenoids. The C-O stretch was shown by the absorption peaks at 1,000 to 1,295. Esters found in plant extracts were the cause.

The H-C-H bend at 1,448.94 suggested the presence of unsaturated alcohols or alkanes in the plant. The absorption peak 1,528.65 suggested an N-H bend caused by steroid amines. The presence of proteins, steroids, lactones, and flavonoids was indicated by the existence of an amide stretch C=O peak at 1,662.44. C=C alkynyl stretch was indicated by the peak at 2126.54. Peaks at 2975.81, 2890.30, and 2834.71 indicated Alkyl C-H stretch caused by glycosides. Peaks at 3620.38, 3779.5, 3829.87, 3874.93, 3892.63, 3908.35, 3925.58, and 3954 revealed that aliphatic compounds exhibit C-H stretching. That could be related to the fatty acid esters present in the plant extracts. The peak at 3779.57 suggested amide N-H stretch, which may have been caused by the plant’s alkaloids (Bibi et al., 2019).

### 3.4 α-Glucosidase inhibition study

The results of α-glucosidase inhibition investigations are summarised in Table 1. AgNPs obtained from *F. cretica* inhibited the α-glucosidase enzyme in a concentration-dependent manner. AgNPs were the most effective at 1,000 μg/mL, suppressing enzymes by 81.74% ± 0.77%. AgNPs had an IC50 value of 250 μg/mL. AgNPs had inhibitory effects comparable to the positive control group. The inhibitory activity of acarbose at the same dose was 91.45% ± 1.50% (IC50s = 42 μg/mL). The inhibitory activity of Ag-NPs at the same concentrations was comparable to that of the standard drug, acarbose.

**TABLE 1:** α-amylase and α-glucosidase inhibition assay results.

α-amylase inhibition results			
Sample	Concentration	%Inhibition	IC_50_
AgNPs from *F. cretica* Sample	1,000	83.53 ± 1.55^ns^	118
	500	72.97 ± 0.50 ^ns^	
	250	65.84 ± 0.33 ^ns^	
	125	56.75 ± 0.66 ^ns^	
	62	51.10 ± 1.00 ^ns^	
Positive Control	1,000	87.72 ± 0.70	40
	500	78.55 ± 0.55	
	250	71.90 ± 1.00	
	125	65.33 ± 1.40	
	62	59.80 ± 0.56	

α-glucosidase inhibition results			
Sample	Concentration (μg/mL)	%Inhibition	IC_50_ (μg/mL)
AgNPs from *F. cretica* Sample	1,000	81.74 ± 0.77**	250
	500	73.84 ± 0.50*	
	250	60.49 ± 1.60*	
	125	51.49 ± 1.90**	
	62	39.50 ± 0.33***	
Positive Control	1,000	91.45 ± 1.50	42
	500	78.85 ± 0.69	
	250	71.33 ± 1.20	
	125	64.89 ± 1.33	
	62	55.90 ± 0.88	

The results of three independent experimental observations were expressed as Mean ± SEM. Following a one-way ANNOVA, a multiple comparison is made. The findings were compared to control groups using the Dunnett’s test. ns: Values not significantly different in comparison to control at the same concentration. *: *p* < 0.05, **p < 0.01 and ***p < 0.001 at the same concentrations.

### 3.5 Study of α-amylase inhibition

AgNPs showed enzyme inhibitory potentials in amylase inhibition assays, and these were compared to the standard drug (acarbose). In investigations on the inhibition of the enzyme α-amylase, all fractions showed a concentration-dependent inhibition, with AgNPs showing the highest percentage inhibitions. At the investigated concentrations of 1,000, 500, 250, 125, and 62 μg/mL, AgNPs demonstrated 83.53 ± 1.55, 72.97 ± 0.50, 65.84 ± 0.33, 56.75 ± 0.66, and 51.10% ± 1.00% enzyme inhibitions, respectively. According to Table 1, IC_50_s for AgNPs were 118 μg/mL. The IC_50_ for the positive control was 40 μg/mL, and the enzyme inhibition was 87.72% ± 0.70%. One of the most effective methods for treating T2DM patients with postprandial hyperglycemia is to inhibit α-glucosidase and α-amylase. Natural products have been shown to be one of the most important sources for developing drugs to treat DM.

Antihyperglycemic effects of many plants have been documented, either on their own or in combination with standard diabetes treatments. Cinnamon, aloe, bitter melon, coffee, guava, cocoa, green tea, nettle, garlic, soybeans, turmeric, and walnuts are only few of the medicinal plants and their fruits and seeds that have received renewed interest for their scientific potential. Phenomenal, galegine, pycnogenol, miglitol, voglibose, and acarbose are only some of the isolated natural compounds used to treat T2DM (Ayaz et al., 2022).

### 3.6 Assay for DPPH antiradicals

AgNPs had a scavenging effect against DPPH radicals in the DPPH anti-radicals assay (Table 2). The percent scavenging potentials of AgNPs were found to be 82.31 (at concentrations of 1,000 μg/mL), 77.92 (at concentrations of 500 μg/mL), 65.96 (at concentrations of 250 μg/mL), 60.37 (at concentrations of 125 μg/mL), and 34.30 (at concentrations of 62 μg/mL) at the same tested concentration. Positive control (ascorbic acid) demonstrated free radical scavenging potentials of 91.50 ± 0.80, 83.59 ± 0.40, 77.35 ± 0.33, 67.80 ± 0.99, and 61.55% ± 0.66% at 1,000, 500, 250, 125, and 62 μg/mL and an IC_50_ of 45 μg/mL, respectively.

**TABLE 2:** DPPH anti-radicals assay results.

Sample Name	Concentration (μg/mL)	%Inhibition	IC_50_ (μg/mL)
AgNPs from *F. cretica* Sample	1,000	82.31 ± 1.55*	210
	500	77.92 ± 0.71^ns^	
	250	65.95 ± 0.38*	
	125	60.37 ± 1.20 ^ns^	
	62	34.30 ± 0.33***	
Positive control	1,000	91.50 ± 0.80	45
	500	83.59 ± 0.40	
	250	77.35 ± 0.33	
	125	67.80 ± 0.99	
	62	61.55 ± 0.66	

Values represent Mean ± SEM of three experimental observations. Ascorbic acid was positive control. ns: values not significantly different when compared with positive control. *p < 0.05 and ***p < 0.001 when compared with control.

### 3.7 Assay for ABTS antiradicals

AgNPs demonstrated a free radical scavenging effect in the ABTS Antiradicals assay. At the investigated doses of 1,000, 500, 250, 125, and 62 μg/mL, the AgNPs was comparably efficient against ABTS radicals, with percent inhibitions of 92.76 ± 0.66, 84.83 ± 0.33, 81.65 ± 0.85, 68.43% ± 0.96% and 65.43% ± 0.89% inhibitions, respectively.

AgNPs had an IC_50_ of 37 μg/mL against ABTS radicals. The AgNPs showed extremely similar percent inhibitions to the positive control, which displayed 94.78 1.60% inhibition at the highest measured concentration (1,000 μg/mL) and an IC_50_ of 9 μg/mL (Table 3). High concentrations (1,000 μg/mL) produced the best inhibitions, while lower concentrations produced less potent results (Table 3).

**TABLE 3:** ABTS anti-radicals assay results.

Sample Name	Concentration (μg/mL)	%Inhibition	IC_50_ (μg/mL)
AgNPs from *F. cretica* Sample	1,000	92.76 ± 0.66 ^ns^	37
	500	84.83 ± 0.33 ^ns^	
	250	81.65 ± 0.85 ^ns^	
	125	68.43 ± 0.96 ^ns^	
	62	65.43 ± 0.89 ^ns^	
Positive control	1,000	94.78 ± 1.60	9
	500	89.33 ± 0.84	
	250	81.55 ± 0.55	
	125	77.00 ± 1.67	
	62	69.90 ± 0.33	

The values show the mean ± standard error of mean of three experimental observations. Positive control was ascorbic acid. ns: values not significantly different when compared with positive control. *p < 0.05 and **p < 0.01 when compared with control.

### 3.8 *In-Vivo* studies

#### 3.8.1 Acute toxicity

Acute toxicity investigation revealed that AgNPs was safe up to a dose of 20 mg/kg body weight because no mortality was seen up to that point. After receiving various doses, the mice were examined for 4 h for various behavioral changes such writhing, drowsiness, photosensitivity, and convulsions. AgNPs was determined to be safe up to 20 mg/kg body weight when varied dosages were given to mice, and no fatalities or morbidities were noticed during 24 h.

#### 3.8.2 Effect of *F. cretica* plant AgNPs on body weight

In Table 4, we can see how AgNPs and metformin led to changes in body weight. Diabetic control mice had a markedly lower final body weight compared to normal control mice. Diabetic mice given nanoparticle treatment experienced a dramatic rise in body weight. Moderate weight gain was observed in the AgNPs treatment group compared to the positive control group. In contrast, diabetic mice given AgNPs showed a significant increase in body weight compared to diabetic control (DC) mice after receiving the treatment, suggesting that the nanoparticles may have a protective effect against the disease.

**TABLE 4:** Comparison of initial and final body weights of the mice of different groups.

Body weights	Group-I (normal-control)	Group-II (diabetic-control-STZ)	Group-III (silver-treated)	Group-V (metformin-treated)
Initial body weights	27.8 ± 1.60	19.2 ± 1.40	37.2 ± 0.8	28.02 ± 0.50
Final body weights	32.2 ± 1.12	15.3 ± 0.50	42 ± 0.7	33.4 ± 1.29

(n = 5 mice per group) The data are presented as Mean ± SEM. For the statistical analysis of the data, one way ANOVA was used. As compared to the groups, all of the results were significantly different (*p* < 0.05). All weights are in gram (g).

#### 3.8.3 Effect of *F. cretica* plant AgNPs on blood glucose

Blood glucose levels were examined in normal and experimental groups of mice on the first, seventh, 14th, and twenty-first days of treatment. When compared to the normal control group, STZ treatment resulted in a significant increase (*p* < 0.05) in blood glucose levels. After 21 days of treatment with AgNPs, there was a significant reduction in blood glucose levels, indicating the hypoglycaemic potential of AgNPs (Table 5).

**TABLE 5:** The Effect of AgNPs of *F. cretica* plant and standard drug Metformin on Pancreas panel: glucose, amylase; Liver function panel, Renal function panel and lipid profile in STZ-induced diabetic mice.

Biochemical parameters (Units)	Group-I (normal control)	Group-II (diabetic control (STZ-Induced mice))	Group-III (silver-treated)	Group-V (metformin-treated)
Blood sugar (mg/dL)	108.6 ± 2.52	190.5 ± 5.0*	109.8 ± 3.39**	124.2 ± 2.20**
S. amylase (U/L)	1949.2 ± 1.51	3440.3 ± 40.04*	1945 ± 2.3**	195.6 ± 0.88**
Serum bilirubin (mg/dL)	0.78 ± 0.06	2.1 ± 0.08*	0.64 ± 0.102**	0.81 ± 0.12**
SGPT (ALT) (U/L)	33.7 ± 0.61	109.3 ± 2.9*	29 ± 0.7**	32.5 ± 1.30**
ALK–Phosphatase (U/L)	167 ± 7.2	287.7 ± 1.73*	153.8 ± 2.51**	163 ± 1.53**
S. albumin (g/dL)	4.5 ± 0.06	2.46 ± 0.04*	3.9 ± 0.05**	4.41 ± 0.05**
Blood urea (mg/dL)	22.3 ± 0.4	48.8 ± 0.3*	25.2 ± 0.5**	32.61 ± 0.5**
S. creatinine (mg/dL)	0.63 ± 0.03	1.81 ± 0.03*	0.574 ± 0.03**	0.75 ± 0.04**
Uric acid (mg/dL)	3.201 ± 0.06	78.011 ± 0.2*	3.764 ± 0.05**	3.001 ± 0.02**
S. cholestrol (mg/dL)	123 ± 1.42	141.5 ± 1.07*	71.6 ± 5.5**	99.8 ± 4.9**
S. triglyceride (mg/dL)	68.9 ± 2.7	75.3 ± 2.4*	64 ± 4.03**	65.5 ± 1.7**
HDL (mg/dL)	37.05 ± 0.49	79.6 ± 0.6*	36.2 ± 0.37**	36.9 ± 0.29**
LDL (mg/dL)	84.2 ± 4.09	127.7 ± 3.90*	69 ± 2.6**	76.3 ± 2.6**

Values are mean ± standard error of the mean (n = 5). ^*^ *P* < 0.05, significantly different with respect to control group;^**^ *P* < 0.05, significantly different with respect to diabetic group (STZ group) for *post hoc* Tukey’s test.

#### 3.8.4 Effects on liver and renal functions tests

The AgNPs treated diabetic mice showed an enhanced level of albumin and decrease level of blood bilirubin, alanine transaminase, and alkaline phosphatase concentrations. There was a significant rise in serum albumin level in the treated diabetic mice as compared to diabetic. The average total albumin concentration in diabetic mice receiving AgNPs treatment was 3.9 ± 0.05 g/dL. The conventional medicine Metformin had a mean total albumin level of 4.41 ± 0.05 g/dL in diabetic mice.

In contrast to the alanine transaminase levels in the untreated diabetic mice, which had a greater mean enzyme activity at 109.3 ± 2.9 U/L, serum alanine transaminase (ALT) or Serum Glutamic Pyruvic Transaminase (SGPT) levels demonstrate a significant reduction in mean activity when treated with AgNPs, from 109.3 ± 2.9 U/L to 29 ± 0.7 U/L. Similar to what was observed in the mice treated with metformin, the AgNPs-treated mice had a highly significant decline in alanine transaminase to close to normal levels. After continued dosing for 21 days, Table 5 demonstrates that alkaline phosphatase significantly decreased to levels close to normal in response to treatment with *F. cretica*’s AgNPs.

The group of mice also receiving metformin showed the greatest reduction in alkaline phosphatase activity, followed by the group getting AgNPs treatment. Serum bilirubin levels were found to be 0.78 ± 0.06 mg/dL in normal mice and 2.1 ± 0.08 mg/dL in diabetic mice produced by STZ, respectively. AgNPs and the common medication Metformin were administered to STZ-induced diabetic mice, and the outcomes were noticeably different from those obtained after STZ administration (Table 5). Table 5 displays the serum urea, creatinine, and uric acid levels, which were relatively high in the diabetic control mice. The levels of urea, creatinine, and uric acid in diabetic mice treated with the nanoparticles decreased significantly over the course of treatment with AgNPs and the standard drug, Metformin and then returned to nearly normal levels.

#### 3.8.5 Histopathological evaluation of pancreas among various groups

**FIGURE 5:**
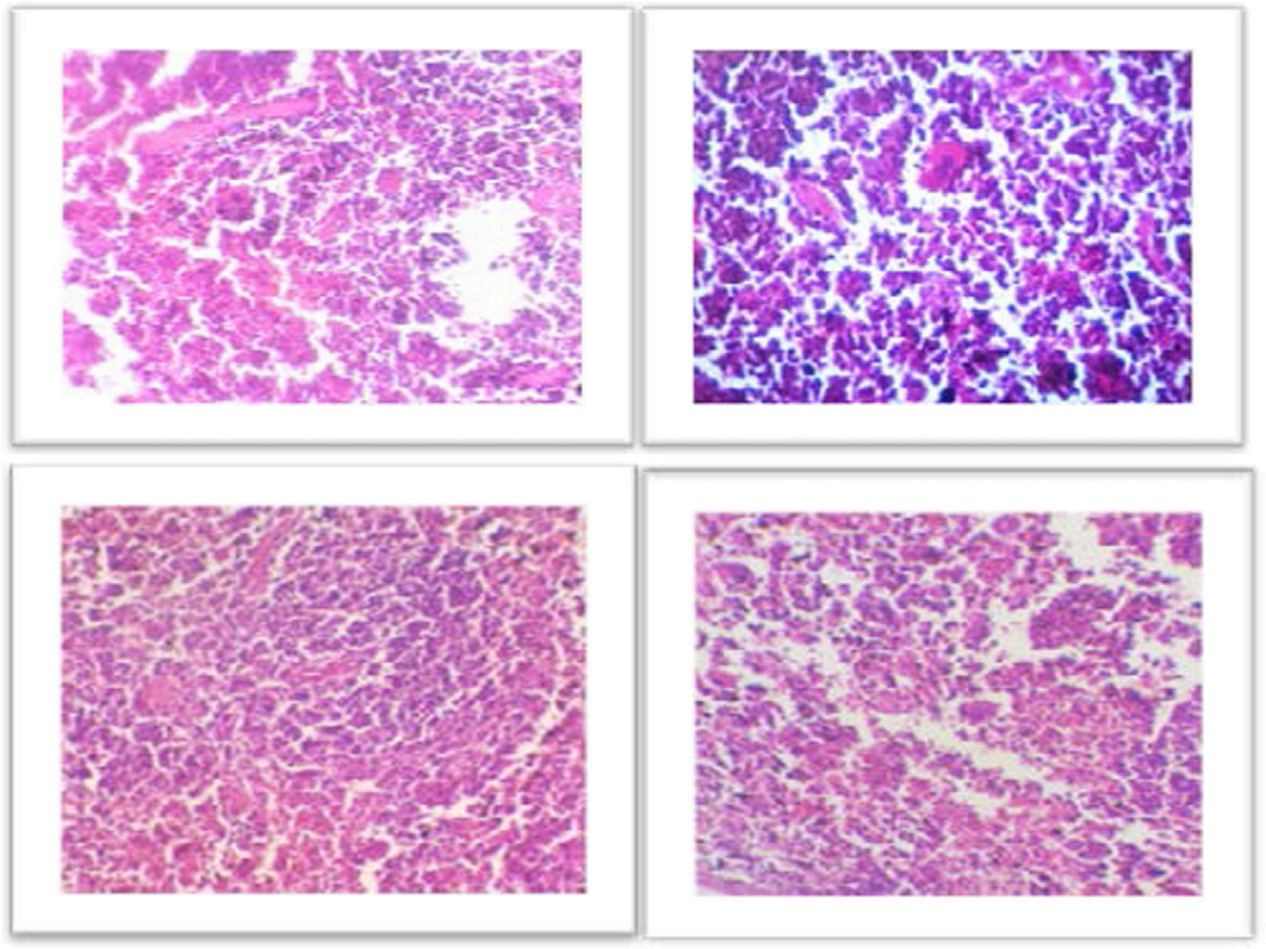
**(A)** Representative images showing Hematoxylin and Eosin stained (H&E) (×40) sections of pancreas. Control, normal pancreas showing normal islets of Langerhans in between normal pancreatic cells. **(B)** Diabetic pancreas showing hyalinized, hypotropic, ruptured and destructed islets of Langerhans with damage in *β*-cells. **(C)** Histopathological slides presenting the effect of AgNPs in streptozotocin-induced diabetic mice’ pancreatic tissues and restores completely the islets of Langerhans. **(D)** Histopathological slides presenting the effect of metformin in STZ-induced diabetic mice’ pancreatic tissues. Some cells are hyalinized, hypotropic and remaining cells are normal.

For the histological evaluation, the pancreas from all four groups were obtained. The result obtained from histological examination clearly determined that the AgNPs regenerate the damaged and necrotic tissues to a greater extent as compared to other groups. To compare the morphology of pancreas of treated and diabetic mice, the results of the H & E stained was observed on ×40 magnification. The pancreatic acini and Islets of Langerhans were observed on 40× in all groups (Figures 5A–D). The Figure 5A showing normal islets of Langerhans in between normal pancreatic cells. The Figure 5B of Diabetic pancreas showing hyalinized, hypotropic, ruptured and destructed islets of Langerhans with damage in β-cells. While Figure 5C showed the istopathological slides presenting the effect of AgNPs in streptozotocin-induced diabetic mice’ pancreatic tissues and restores completely the islets of Langerhans. And the Figure 5D showing histopathological slides presenting the effect of metformin in STZ-induced diabetic mice pancreatic tissue.

## 4 Discussion

Since ancient times, medicinal plants have been used, and in recent years, drugs derived from plants and other natural sources have become more common. Finding treatments from plants as well as alternative sources is of great interest to pharmacologists (Kamran et al., 2017). Researchers are paying a lot of attention to the synthesis of functional nanomaterials because of their many potential uses in fields like biomedicine, cancer treatment, bio-imaging, drug delivery, molecular-based detection, *etc.* However, the physical and chemical methods used to synthesize nanomaterials produce massive quantities of hazardous chemicals and toxic by-products, which have serious consequences for both the environment and human health. The development of non-toxic, safe, effective, non-lethal, environmentally friendly, and economically viable biological methods for the synthesis of NPs is therefore of increasing importance. Numerous recent reports on the synthesis of AgNPs have made use of leaf, stem and root extracts from plants such as *Eucommia ulmoides*, *Dracaena cochinchinensis, Gloriosa superba, Sargassum polycystum* and *Raphanus sativus* (Zulfiqar et al., 2019). AgNPs are the most widely used of these particles due to their wide range of applications.

The *F. cretica* is well known as a medicine. The subject plant was found to be effective against fever, toothache, asthma, scabies, stomach problems, tumors, and urinary discharges. It was also said to have antimicrobial, anti-inflammatory, anti-bleeding, thrombolytic, and antioxidant properties (Kiani et al., 2022). Very little is known about how *F. cretica* plant extracts can be used to make AgNPs.

*F. cretica*’s bioreducing agents used to synthesize AgNPs are of great interest due to the NPs’ novel properties. As a result of the excitation of the NP surface plasmons, the reduction of AgNO_3_ by the *F. cretica* extract results in a change in color from light orange to dark red, indicating a significant absorption of visible light. The Ag NPs’ size, shape, and concentration all affect how the color changes (Wang and Joseph, 1999). Moreover, UV-vis spectra were taken to confirm the reduction of Ag ions to metallic AgNPs, and the maximum absorbance was seen at 205.5, 206.5, and 211.5 nm (Figure 1). Bogle et al. (Bogle et al., 2006), reported the UV-spectra of AgNPs around 455 nm, Banala reported at 470 nm (Banala et al., 2015), Ali et al., reported (Ali et al., 2023) UV-spectra at 420 nm. The size of NPs is a major concern, especially for their use in biology, because the size of NPs has a big effect on how fast they move through biological membranes. For specific biological applications, it is preferable that NPs be of a certain size; Nevertheless, it has been noted in the past that smaller NPs have greater penetrating capability; yet, the problem with having a size that is too small is that it brings the issue of higher toxicity in comparison to bigger size NPs (Hasan et al., 2013). Prior to their biological application, TEM and SEM were used to analyze the morphology, size, and form of NPs. Since the manufactured NPs are below the safe threshold for causing toxicity within cells, the several NPs with an average size of 29.39 nm are ideally suited for biomedical applications. According to Ul Haq, the spherical and mono-dispersed NPs generated by the green-mediated synthesis of AgNPs using *P. niveum* methanolic extract ranged in size from 12 to 28 nm (average: 21 nm) (Haq et al., 2022). Nagaraja reported the generated AgNPs had sizes ranging from 52.12 nm to 65.02 nm (Nagaraja et al., 2022). The spherical, rhomboid, and cubical AgNPs produced with the use of Punica granatum leaf extract had an average size of 98.93 nm and a diameter that ranged from 88.0 to 120 nm (Kumar et al., 2018). The size of alion-mediated produced nanoparticles, according to Kumar et al. (Kumar et al., 2013), was in the range of 287–293 nm, with an average size of 70 nm. According to Firdhouse et al. (Firdhouse et al., 2012), AgNPs made from ethanolic leaf extract ranged in size from 20 to 150 nm.

Figure 3’s TEM picture of AgNPs reveals that the nanoparticles are spherical. Previous TEM analysis conducted by Ul Haq (Haq et al., 2022), Bibi (Bibi et al., 2019), Nadzir (Nadzir et al., 2019), Zulfiqar (Zulfiqar et al., 2019), Ibrahim (Ibrahim, 2015) and Kumar (Karunakaran et al., 2017) also demonstrated that AgNPs have a spherical form. There is conclusive evidence that the particle size was less than 100 nm, confirming the presence of nanoparticles. Although the antibacterial capabilities of AgNPs are reliant on their size, spherical nanoparticles with lower sizes demonstrate superior physical qualities. Dark colours on the AgNPs indicated the presence of secondary elements, which could be biocomponents derived from plant extracts (PE). These biocomponents are essential for the promotion of Ag + reduction to AgNPs. In addition, these particles serve as a capping agent that prevents agglomeration. According to the Fathi et al. (Fathi et al., 2017), SEM showed that the nanoparticles were all the same size (20–30 nm) (Shankar et al., 2004). did a study on how to make AgNPs using Azadirachtaindica. The results of this study are the same as what they found. Nanoparticles in the study were between 5 and 35 nm in size and had a round shape (Sivaraman et al., 2009). The size of AgNPs was reported to be 10 nm by (Ahmad et al., 2010) and 15 nm by (Chandran et al., 2006).

According to Nagaraja et al. (Nagaraja et al., 2022), FT-IR research shows that *Psidium guajava* extract (PGE) contains numerous functional groups that reduce and stabilize NPs before production. FT-IR spectrum was 4,000–500 cm^−1^. Alkane C-H stretching vibration mode is at 2977 cm^−1^. PGE primary amines (proteins) are at 1,582 cm^−1^. Phenols’ O-H bond is the peak at 1,388 cm^−1^. FT-IR measurements of the plant extract showed active biomolecules that capped and reduced Ag^+^ to Ag^0^ (Khan et al., 2020). Souha et al. (Souha et al., 2021) found that the diabetic group that got AgNPs made either with a chemical method or with Eryngium extract had much lower levels of glucose, urea, ALT, and the relative gene expression of chemerin and resistin than the diabetic control group. The amount of creatinine in the blood did not change much in either the control diabetic group or the diabetic group that got SNPs made chemically or with the Eryngium extract. SNPs that are made chemically or in a green way protect liver hepatocytes from damage. They decreased inflammatory factors by lowering blood sugar. Diabetes Mellitus (DM) often causes dyslipidemia, which raises cholesterol and triglycerides. Hormone-sensitive enzymes like lipase may cause this rise (Soni et al., 2018). Hypertriglyceridemia and hypercholesterolemia (Ayaz et al., 2022) are the most prevalent lipid disorders in diabetics. In our work, STZ-induced diabetic mice had greater levels of cholesterol and triglyceride than AgNPs and Metformin-treated mice. Nagaraja (Nagaraja et al., 2022) found that PGE and *Psidium guajava* (PG) AgNPs significantly lowered blood glucose in diabetic rats, avoiding weight loss and improving lipid profile markers. PGE and PG AgNPs improved pancreas and liver cells histopathologically. Ul Haq (Haq et al., 2022) stated there was a dramatic decrease in blood glucose levels, a rise in body weight, and a marked enhancement in lipid, liver, and kidney profiles. Levels of total cholesterol, triglycerides, HDL, and LDL in the blood were affected by *Phagnalon niveum* extracts and AgNPs. Cholesterol, triglyceride, and high density lipoprotein (HDL) levels all rose in alloxan-induced diabetic rats. Fluctuating blood lipid levels are definitely caused by extended cyclic adenosine monophosphate, which is responsible for lipid formation. AgNPs significantly decreased cholesterol, triglyceride, and LDL levels while increasing HDL levels in diabetic rats. There was a notable correction of anomalies in body weight, urine, and serum levels after administration of the biosynthesized AgNPs, suggesting the drug has great potential as an anti-diabetic treatment.

Wahab (Wahab et al., 2022) reported that the goal of the study is to shed light on the therapeutic potential of AgNPs as an antidiabetic drug in STZ-induced diabetic BALB/C mice by reporting an unique synthesis of these nanoparticles utilizing aqueous extract of *Thymus serpyllum*. The α-amylase inhibition and antioxidant activity were checked through *α-*amylase and DPPH radical scavenging assay, respectively. AgNPs mediated by Thymus serpyllum possess substantial antioxidant and anti-diabetic properties and can be researched further as a cost-effective alternative treatment for T2DM. According to our previous study, the SeNPs of *F. cretica* exhibited good anti-diabetic, antioxidant activities as well as anti-hyperlipidemic via both* in-vitro* and *in-vivo* approaches (Khan et al., 2023). So, our results of AgNPs correlate well with the literature surveys.

## 5 Conclusion

According to the findings of the current research, the AgNPs that were derived from the leaf extract of *F. cretica* demonstrated properties that were anti-oxidant, anti-hyperlipidemic, and anti-diabetic. Additionally, they have a protective effect on the kidneys and the liver. The findings of the study were evaluated *in-vitro* and *in-vivo* using methods that are intended to treat diabetes. It was discovered that the AgNPs that were generated from the leaf extract might have qualities that could help treat diabetes. Therefore, the AgNPs found in leaf extract can be utilised to protect tissues from oxidative stress, which is possibly the root cause of the glycemic effects of the extract. AgNPs, which are obtained from leaf extract, might 1 day be manufactured on a commercial scale and put to use in the treatment of diabetes. AgNPs of the *F. cretica* have been shown to have substantial anti-oxidant and anti-diabetic properties and may be further investigated as a useful and less expensive treatment option for T2DM.

## Data Availability

The raw data supporting the conclusion of this article will be made available by the authors, without undue reservation.
